# A review and update of the Health of the Nation Outcome Scales (HoNOS)

**DOI:** 10.1192/bjb.2017.17

**Published:** 2018-04

**Authors:** Mick James, Jon Painter, Bill Buckingham, Malcolm W. Stewart

**Affiliations:** 1 Royal College of Psychiatrists, London, UK; 2 Sheffield Hallam University, Sheffield, UK; 3 Australian Government Department of Health, Canberra, Australia; 4 Thrive Psychology, Auckland, New Zealand

## Abstract

**Aims and method:**

The Health of the Nation Outcome Scales (HoNOS) and its older adults’ version (HoNOS 65+) have been used widely for 20 years, but their glossaries have not been revised to reflect clinicians' experiences or changes in service delivery. The Royal College of Psychiatrists convened an international advisory board, with UK, Australian and New Zealand expertise, to identify desirable amendments. The aim was to improve rater experience by removing ambiguity and inconsistency in the glossary rather than more radical revision.

**Results:**

Changes proposed to the HoNOS are reported. HoNOS 65+ changes will be reported separately. Based on the views and experience of the countries involved, a series of amendments were identified.

**Clinical implications:**

While effective clinician training remains critically important, these revisions aim to improve intra- and interrater reliability and improve validity. Next steps will depend on feedback from HoNOS users. Reliability and validity testing will depend on funding.

**Declaration of interest:**

None.

The Health of the Nation Outcome Scales (HoNOS)^1^ is a 12-scale clinician-rated measure developed by the Royal College of Psychiatrists to guide everyday clinical practice and measure health and social care outcomes in secondary care mental health services for working-age adults (18–65 years). The HoNOS was designed to:

(1) be short and simple for routine use and acceptable to a range of mental health professionals
(2) have adequate coverage of clinical and social functions
(3) be sensitive to improvement, deterioration or lack of change over time
(4) have demonstrable and acceptable reliability
(5) have a known relationship with more established scales.^2^

Over its 20-year lifespan, the HoNOS has demonstrated adequate reliability, validity, clinical utility^2^^,^^3^ and sensitivity to change.^4^^,^^5^ Subsequent to its development, a family of related measures have been developed for different age groups and clinical populations.^6^^–^^8^ The HoNOS has been translated into at least 12 other languages and is routinely used in clinical practice and research around the world. England, Australia and New Zealand, have also mandated the HoNOS for routine monitoring and outcome measurement across their mental health services at a national level.^9^^–^^12^

Although the basic soundness of the instrument is recognised, two decades of training, routine use and analysis of the resultant data, together with substantial advances in psychiatry and mental health services, have shown that updates to the supporting documentation are required to improve use of the HoNOS.^3^^,^^4^^,^^13^ As copyright holder for the HoNOS family of measures, the Royal College of Psychiatrists elected to undertake a limited review (rather than a full redevelopment and revalidation) that aimed to use expert opinion to improve the utility of the HoNOS in contemporary mental healthcare, while remaining true to its original aims and maintaining comparability with existing HoNOS data-sets. This paper outlines the review process, its scope, the issues identified and the set of revised scales.

## Method

In recognition of each country's mandated use of HoNOS at a national level,^9^^–^^12^ and to ensure their interests were incorporated, an advisory board (chaired by the Royal College of Psychiatrists' national HoNOS advisor) was drawn from representatives from England, Australia and New Zealand. Nominations from the respective national governments were made, with members being required to have extensive experience in either: HoNOS staff training; using HoNOS in practice; using HoNOS data at a macro level; or providing oversight at service, professional or governmental level.

Advisory board members were asked to use their professional networks to canvas widely for clinicians' opinions regarding aspects of the tool that required refinement. To facilitate this, a standard recording form, covering each scale (as well as the overall rating guidelines) was provided, which, when collated, acted as a review template (Table 1). The board also considered the evidence and recommendations previously prepared in the review by Trauer and Buckingham^3^ commissioned by the Australian Government Department of Health.

**Table 1:** Summary of issues raised

Scale/Section	Issues recommended for consideration
Overall rating guidelines	• Need additional guidance about incorporating cultural factors into ratings • Improve clarity of the scoring system in relation to clinical significance • Improve clarity regarding what is to be rated (i.e., most severe problem or usual level of difficulty in past 2 weeks) • Clarity about when the scales should be used
Scale 1. Overactive, aggressive, disruptive or agitated behaviour	• Ensure all four behaviours are considered and clarify how many need to be present • Clarify to what extent behaviours here should be related to mental health problems • Provide guidance about how to address relevant cultural factors and context • Consider adding expansive mood as underrepresented by Scale 8
Scale 2. Non-Accidental self-injury	• How to address relevant cultural factors and context • Clarify that this is not an assessment of risk • High-risk thoughts/intentions currently underrated relative to overall severity • Increase consistency of dimensions of risk considered at different levels.
Scale 3. Problem drinking or drug-taking	• Provide clarity on the rating of tobacco use • Clarify how behaviours associated with addiction are rated • Improve clarity of rating for binge drinking and the reference to social norms that can lead to subjective rating • Clarify how ratings should take into consideration people on substitution programmes (e.g. methadone)
Scale 4. Cognitive problems	• Clarify where formal thought disorder and a lack of insight should be rated • Perceived to be a large gap in severity between rating 2 and rating 3 anchor points • Descriptors focus primarily on dementias, not other cognitive difficulties • Review problems associated with transient versus enduring cognitive impairments within this scale
Scale 5. Physical illness or disability problems	• Glossary descriptions reported by many to be unclear and/or unhelpful, especially in comparison with the HoNOS 65+ descriptors
Scale 6. Problems associated with hallucinations and delusions	• Clarify where body image disturbance related to eating disorders should be rated • Improve description of ‘odd or eccentric ideas’ • Clarify where ‘lack of insight’ should be rated
Scale 7. Problems with depressed mood	• Clarify that this scale is about depressed mood rather than clinical depression, as other symptoms of clinical depression cause confusion in the field • Change examples used to clarify ratings, as these were not found to be helpful in the field (e.g. guilt or self-accusation)
Scale 8. Other mental and behavioural problems	• Consider whether any changes could be made that would retain the current features of the scales while addressing the high levels of use of labels A and D in this scale • Relatively poor reliability for this scale • Consider the addition of an option for elated mood, as this is not represented elsewhere in the scales • Clarify whether stress should relate to general life stress or specifically acute stress reaction and post-traumatic stress disorder • Clarify where body image disturbance should be rated
Scale 9. Problems with relationships	• Improve clarity about whether clinicians should score worst or usual level of relationship difficulties • Improve glossary examples to better ensure full range of relationship difficulties identified (e.g. destructive or unhelpful relationships, active or passive withdrawal)
Scale 10. Problems with activities of daily living	• Improve instructions on how to combine assessment of deficits in basic and complex skills into a single rating • Clarify how clinicians should determine the effects of existing supports • Review and evaluate the perceived disproportionate jump in severity from rating 2 to rating 3
Scale 11. Problems with living conditions, and Scale 12. Problems with occupation and activities	• High missing data rates from in-patient settings • Provide additional clarity regarding the use of the 2-week rule for these scales • Review the perceived inconsistencies between the descriptors for the different levels of severity • Provide more formal clarification about how to rate these scales for long-term in-patients and residential settings
Other matters	• Review the terms used for patients, staff and carers • Explore the feasibility and desirability of trying to build consistency between the HoNOS and the HoNOS 65+

This review template formed the basis of a scale-by-scale review. Some suggestions for change were more radical than others; hence, the board developed criteria with which to gauge their viability. For a change to be supported, it needed to represent a tangible improvement (e.g. removal of anachronisms or ambiguities, or simplifying the instrument's use) whilst also:

(1) maintaining the original instrument's integrity as much as possible
(2) ensuring that individual and aggregated outputs were likely to remain comparable with existing data
(3) supporting HoNOS as a summary of clinical assessment(s)
(4) adhering to the HoNOS ‘core rules’ i.e.
  • each item is a behaviourally anchored 5-point scale
  • rate items in order (1–12)
  • use all available information to make a rating
  • do not include information already rated in an earlier item
  • rate the most severe problem/worst manifestation from the preceding 2 weeks
  • a problem is rated according to the degree of distress caused and/or its effects on behaviour
  • must be rated by a mental health professional trained in clinical assessment
  • rate problems regardless of cause.^2^

Some changes identified had consequences/implications for other items, and hence an iterative process of minuted teleconference and email discussions evolved, between October 2014 and January 2016.

Following review of the HoNOS documentation, a review of HoNOS 65+ was also undertaken through to October 2016. This presented an opportunity to maximise alignment between the two versions of the instrument and yielded a number of additional refinements to both measures.

## Results

After working through the issues set out in Table 1, and reviewing the HoNOS 65+, the advisory board produced a set of revised HoNOS scales (supplementary Table 1 available at https://doi.org/10.1192/bjb.2017.17). Each item's original wording is also included (in greyed-out boxes) to aid comparison.

## Discussion

### Overarching HoNOS rating guidelines

Despite the agreed objective of keeping the instrument short and easy to use, based on considerable experience of training and routine clinical use, advisory board members universally agreed that the original rating instructions erred on brevity at the expense of clarity. They also recognised that the frequency, duration and quality of training varies significantly.^3^^,^^14^ Therefore, to improve interrater agreement (but not to replace formal training), existing training materials and protocols were reviewed and, in many cases, incorporated.

The first notable augmentation was to legitimise the informal training advice that ratings of 0–1 should be viewed as subclinical, whereas ratings of 2–4 indicate problems of a severity that would normally warrant care/treatment planning and intervention.

Second, the original guidance stipulated that behaviours/problems should be rated regardless of cause (i.e. irrespective of psychiatric disorder) but was silent on the rating of issues deemed normal in an individual's culture/subculture. Issues for which the ratings may be affected by cultural and contextual factors have been previously identified^3^ and include culturally sanctioned aggression (Scale 1), self-harm associated with religious ceremonies or periods of mourning (Scale 2), paranormal experiences associated with cultural beliefs or events (Scale 6), and the expression of sadness associated with bereavement (Scale 7). None of these are attributable to mental health problems and, if rated, would produce a misleading clinical picture. Therefore, although cultural competence remains a prerequisite to good-quality clinical assessment (and thus accurate ratings), there is now an explicit expectation that an individual's culture should be taken into account. This debate also raised a wider question for the advisory board about how attributable to mental ill health behaviours needed to be before they should be included, an issue that was carried into amendments to several individual scales.

Consideration was given to what terms should be used to describe people who use mental health services, their significant others, and staff. This, in part, reflected moves from the recovery perspective and the mental health consumer movement to minimise the extent to which language used is pathologising and pejorative.^15^ Discussion indicated that terminology varied between countries, over time and between groups within countries. Given the lack of consistency, the decision was made to retain the term patient to denote a person who uses mental health services, family for people who are significant others of that person, and staff for people who are paid to provide mental health services.

#### Scale 1 Overactive or aggressive or disruptive or agitated behaviour

It was recognised that while item 1 has a broader scope than most others, clinicians focus primarily on the aggressive elements of the scale.^3^ The case for creating a separate item for this aspect alone was judged to be a more fundamental change than the current review's scope could support. Instead, the item description was revised to emphasise the need to consider all four aspects. The issue of culturally sanctioned aggression in the context of ritual was felt to have been addressed in the overarching rating guidance, hence the scale remaining diagnosis-agnostic.

#### Scale 2 Non-accidental self-injury

Revisions here were intended to provide consistency of examples across the severity ratings (covering risks and thoughts as well as behaviours). Cultural influences (e.g. ritual self-flagellation commonplace in some religions^16^) continue to require a culturally competent clinician and reference to the overarching guidance.

#### Scale 3 Problem drinking or drug-taking

As with Scale 2, changes now provide consistent descriptions of key elements of addictive behaviours, with each level describing aspects of craving, dependency and behaviour that align to contemporary notions of severity (e.g. National Institute for Health and Care Excellence guidance^17^). The more subjective aspects of the original scales (e.g. ‘within social norms’ and ‘loss of control’) have been removed, and there is an increased emphasis on the psychological effects of drug and alcohol use. This ensures that, during periods of enforced abstinence (e.g. hospital admissions), the severity of addiction can still be captured.

Finally, the advisory board, while fully acknowledging the harmful effects of tobacco use,^18^ agreed to explicitly exclude smoking from this scale – a significant decision that warrants further explanation. First, as per the original text, the physiological consequences of smoking will continue to be captured by Scale 5. Second, the prevalence of smoking in people with mental health conditions is approximately twice the norm,^19^ creating a ‘shadowing effect’ that can detract from the scale's clinical utility. There are, of course, more extreme scenarios where, for example, individuals render themselves vulnerable to exploitation through their attempts to obtain cigarettes. The new guidance therefore excludes dependence on tobacco unless there are severe and adverse consequences above and beyond the known detrimental effects to physical health.

#### Scale 4 Cognitive problems

Feedback suggested that Scale 4 was too heavily orientated towards dementia and, even then, some of the examples were deemed unhelpful. Revisions were therefore undertaken in two stages. Initially, with reference to other versions of HoNOS,^8^^,^^20^ the narrow focus on dementia was broadened to incorporate issues such as formal thought disorder and the ability to learn. Through this process, the reported ‘excessive jump’ between ratings of 2 and 3 was also addressed. Then, in parallel with the HoNOS 65+ review, the descriptions were adjusted further. This led to improved alignment between the HoNOS and HoNOS 65+ cognitive scales, but complete alignment was regarded as too radical a change.

#### Scale 5 Physical illness or disability problems

No changes to this scale were deemed necessary.

#### Scale 6 Problems associated with hallucinations and delusions

This scale only required minor linguistic changes.

#### Scale 7 Problems with depressed mood

The descriptors for ratings 2–4 are now consistent with the scale title (i.e. depressed mood rather than depression), thus removing ambiguity surrounding the inclusion/exclusion of other depressive symptoms. This point has also been reiterated in the scale's initial bullet points. Training experience has shown that the original descriptors led clinicians to focus heavily on the concept of guilt at the expense of other manifestations of low mood. Consequently, as for Scale 1, a more consistent and balanced description of each severity rating (in this case including loss of interest, guilt and loss of self-esteem) has been created.

#### Scale 8 Other mental and behavioural problems

The frequency with which anxiety is rated within this scale^3^^,^^10^ has resulted in calls for its promotion to that of a scale in its own right. While this proposal has merit, it was deemed a substantial change and thus out of scope for inclusion in this work. The possibility of rating multiple issues on this scale was also discussed, but would again affect comparability with existing data, contradict the ‘rate the worst’ rule and overly complicate the rating guidance for relatively little benefit. As a result, these two proposals were reserved for a more extensive review should the opportunity arise.

HoNOS trainers reported frequently being asked where elated mood should be rated. There was a suspicion that it was often captured under the ‘other’ option in this scale, or rated by proxy in Scale 1 (although no empirical evidence was available). To improve consistency of rating, it was introduced as a specific option (‘K’) in Scale 8. (N.B. The letter J has not been reused to avoid potential confusion between data-sets collated from the use of the original HoNOS and this revised version).

Based again on training experiences, while options A–I have been retained, each has been supplemented with explanatory text. DSM-5^21^ was the genesis for these additions, but descriptors have been heavily edited to ensure they described presenting needs/problems rather than merely reflecting diagnostic criteria. This clearly challenged the core principle of brevity but was felt to be outweighed by the benefits arising from improved clarity.

#### Scale 9 Problems with relationships

Changes to this scale were limited to modest rewording of descriptions, again intended to increase clarity.

#### Scale 10 Problems with activities of daily living

Additional introductory text has been added to reflect common training advice regarding how to ‘manage’ the effects of any existing support the patient is receiving. The considerably more complex and granular approach that might be required to accommodate the occasional problems experienced when rating patients whose complex skills are intact, but whose self-care skills are not, was also considered. The approach used by the tabulated version of the HoNOS 65+^22^ was suggested as a possible model to accommodate this, but was deemed to represent a substantial change and hence rejected.

#### Scale 11 Problems with housing and living conditions, and Scale 12 Problems with occupation and activities

The issues and solutions for the final two scales were very similar, and hence their discussion has been combined. First, experience from the field suggested that, without adequate training, Scales 11 and 12 are often used to consider aspects of the patient's abilities (as in Scale 10) rather than to rate how well their current environment matches their needs in terms of accommodation or occupation and activity. Alternatively, these scales can be misused as global ratings of the quality of accommodation and occupation/activity. Either way, the conceptual complexity causes difficulty in routine use.^23^ An additional bullet point now addresses these misconceptions.

Second, in recognition of the rating difficulties that can arise at/around the point of hospital admission and discharge,^24^ a thorough review of supplementary advice provided in each country was undertaken. The option to vary the 2-week rating period for these scales was considered, but the board was uncomfortable breaching this core rule. Instead, a less radical solution was to highlight that ‘the patient's usual ….’ was to be rated and provide clearer guidance about how this should be dealt with in different living situations. Retaining this degree of clinical discretion was deemed both tolerable and more likely to result in clinically meaningful ratings.

Other revisions to these scales were less complex and primarily sought to update some of the terminology used and ensure all terms were acceptable to each participating country.

### Other issues

In addition to the changes discussed, the review highlighted further areas for development that may be considered desirable. However, these constitute substantial changes that fall outside the scope of the agreed review. These would require the development of a new instrument but remain an option for future development pending sector agreement, as well as government interest and funding.

### Implications

The Council of the Royal College of Psychiatrists considered the proposed changes to the HoNOS set out in this paper at its meeting on the 14 July 2017 and agreed to these recommended changes proposed by the advisory board. In doing so, the Council acknowledged that it is highly desirable that the perceived benefits of the changes be subjected to empirical testing through assessment of interrater reliability and revalidation of the measure in the field. Such testing will require funding and ideally the involvement of those countries that have heavily invested in the HoNOS to date; this is being pursued by members of the advisory board.

It is also acknowledged that there are likely to be issues that will affect the implementation of a revised version in the different jurisdictions involved in the review, as well as in other parts of the mental health community worldwide that have invested in the use of the HoNOS and translations of the original scales. One such effect might be on the programmes of training for clinicians; while the proposed changes are intended to improve the clinician experience of using the scales, they do not obviate the need for training in the use of the scales.

## About the authors

**Mick James** is the National HoNOS Advisor and MHCT (Mental Health Clustering Tool) Project Manager at the Royal College of Psychiatrists, London, UK; **Jon Painter** is Joint Clinical Lead of the Care Pathways and Packages Project, National Health Service England, and is a Mental Health Nurse Lecturer at Sheffield Hallam University, Sheffield, UK; **Bill Buckingham** is a Consultant and Mental Health Technical Advisor to the Australian Government Department of Health, Canberra, Australia; and **Malcolm W. Stewart** is a Consultant Clinical Psychologist at Thrive Psychology, Auckland, New Zealand.
